# Computer mouse use captures ataxia and parkinsonism, enabling accurate measurement and detection

**DOI:** 10.1002/mds.27915

**Published:** 2019-11-07

**Authors:** Krzysztof Z. Gajos, Katharina Reinecke, Mary Donovan, Christopher D. Stephen, Albert Y. Hung, Jeremy D. Schmahmann, Anoopum S. Gupta

**Affiliations:** ^1^ Harvard John A. Paulson School of Engineering and Applied Sciences Cambridge Massachusetts USA; ^2^ Paul G. Allen School of Computer Science and Engineering University of Washington Seattle Seattle Washington USA; ^3^ Department of Neurology, Massachusetts General Hospital Harvard Medical School Boston Massachusetts USA; ^4^ Ataxia Unit, Department of Neurology Massachusetts General Hospital, Harvard Medical School Boston Masssachusetts USA; ^5^ Movement Disorders Unit, Department of Neurology, Massachusetts General Hospital Harvard Medical School Boston Massachusetts USA

**Keywords:** ataxia, clinical trials, machine learning, outcome measures, parkinsonism

## Abstract

**Background:**

Objective assessments of movement impairment are needed to support clinical trials and facilitate diagnosis. The objective of the current study was to determine if a rapid web‐based computer mouse test (Hevelius) could detect and accurately measure ataxia and parkinsonism.

**Methods:**

Ninety‐five ataxia, 46 parkinsonism, and 29 control participants and 229,017 online participants completed Hevelius. We trained machine‐learning models on age‐normalized Hevelius features to (1) measure severity and disease progression and (2) distinguish phenotypes from controls and from each other.

**Results:**

Regression model estimates correlated strongly with clinical scores (from *r* = 0.66 for UPDRS dominant arm total to *r* = 0.83 for the Brief Ataxia Rating Scale). A disease change model identified ataxia progression with high sensitivity. Classification models distinguished ataxia or parkinsonism from healthy controls with high sensitivity (≥0.91) and specificity (≥0.90).

**Conclusions:**

Hevelius produces a granular and accurate motor assessment in a few minutes of mouse use and may be useful as an outcome measure and screening tool. © 2019 The Authors. *Movement Disorders* published by Wiley Periodicals, Inc. on behalf of International Parkinson and Movement Disorder Society.

Drug development efforts are underway for patients suffering from neurodegenerative diseases, including cerebellar ataxias, Parkinson's disease (PD), and Parkinson‐plus syndromes. Key challenges for clinical trials include the ability to accurately diagnose early disease^1^, ^2^, ^3^, ^4^ and confidently measure disease change. These challenges arise in part because current assessments of neurodegenerative diseases are subjective, exhibit intra‐ and interrater differences,^5^ and are poorly accessible because they have to be performed in a clinical setting by a movement disorders specialist.

Such challenges are amplified in children in whom norms for movement evolve rapidly with age. Furthermore, disease‐tailored clinical scoring scales are limited in their ability to measure nonprototypical phenotypes, for example, in ataxia patients with bradykinesia. Because of the complex, heterogeneous, and overlapping phenotypes in neurodegenerative diseases, it would be advantageous to complement existing assessment methods with a readily available tool that could characterize movement across a number of phenotypes.

We have developed a rapid, computer mouse–based tool called Hevelius that quantifies arm function by extracting 32 features from continuous, target‐driven computer mouse trajectories (see Supplementary Methods for task and analysis details). Here, we demonstrate the effectiveness of Hevelius (1) to accurately measure disease severity and (2) to distinguish patients with ataxia or parkinsonism from controls and from each other.

## Results

### Participant Demographics

Data from 229,017 online participants were used to develop the normative data set. Participants self‐reported coming from 158 countries, with the largest group coming from the United States (43.8%).

One hundred and eighty‐nine patients were assessed using Hevelius in the clinic setting: 95 with cerebellar ataxia, 46 with parkinsonism, and 29 controls (see Table 1). Eighteen individuals with a progressive ataxia diagnosis (12 with spinocerebellar ataxia [SCA], 4 with ataxia‐telangiectasia [A‐T], and 2 with multiple system atrophy, cerebellar‐type [MSA‐C]) completed the task at an additional point. For mixed movement disorders such as MSA, we relied on the treating neurologist's assessment to group the individual into ataxia versus parkinsonism. The dominant arm was equally or more affected than the nondominant arm in 82 of 141 individuals with ataxia or parkinsonism. Individuals with neurologic disease (median, 3.1 minutes) took longer than healthy controls (median, 1.9 minutes) to complete the task (F_1,185_ = 19.99, *P* < 0.0001).

**Table 1:** Participant demographics

	Online	Clinical		
	Controls	Controls	Ataxia	Parkinsonism
n	229,017 (total) Ages 14‐62: >1000 each Ages 11‐76: >100 each	29	95 (total) 28 SCA, 10 A‐T, 6 MSA‐C, 6 HSP, 4 AIA, 2 EA2, 2 ARCA1	46 (total) 39 idiopathic PD, 7 atypical parkinsonism
Age	5–85 (M ± SD 33.2 ± 12.4)	8–60 (M ± SD 25.6 ± 13.2)	7–78 (M ± SD 51.5 ± 19.3)	45–82 (M ± SD 66.1 ± 7.7)
Sex	65.5% male, 33.3% female, 1.2% not given	58.6% male, 41.4% female	56.8% male, 43.2% female	73.9% male, 26.1% female
Handedness		96.6% right, 3.4% left	94.7% right, 5.3% left	89.1% right, 10.9% left
Disease severity (dominant arm clinical score on BARS or UPDRS)			BARS (scale, 0–4): 0–3 (M ± SD 1.0 ± 0.7)	UPDRS composite (scale, 0–24): 0–11 (M ± SD 3.8 ± 2.6)
Disease severity (overall clinical score on BARS or UPDRS)			BARS (scale, 0–30): 0–23.5 (M ± SD 10.4 ± 5.1)	UPDRS part III (scale, 0–108): 1–51 (M ± SD 16.9 ± 9.5)

Note: in all analyses throughout the article, age differences between groups were adjusted for through the age‐specific *z*‐scoring process described in the Methods section.

M, mean; SD, standard deviation; UPDRS, Unified Parkinson's Disease Rating Scale; BARS, Brief Ataxia Rating Scale; SCA, spinocerebellar ataxia; A‐T, ataxia‐telangiectasia; MSA‐C, multiple system atrophy, cerebellar‐type; HSP, hereditary spastic paraplegia; AIA, autoimmune‐related ataxia; EA2, episodic ataxia type 2; ARCA1, autosomal recessive cerebellar ataxia type 1; PD, Parkinson's disease.

“UPDRS composite” score (range, 0–24) is defined as the sum of the UPDRS arm subscores: rest tremor (0–4), postural tremor (0–4), rigidity (0–4), and bradykinesia on 3 tasks (0–12).

### Summary Statistics for Online Participants

Supplementary Figure S3 (top) shows how 4 representative measures collected by Hevelius varied across the life span in the cross‐sectional sample collected online. As expected, basic aspects of performance, such as overall efficiency (measured by movement time) or the ability to control movement speed (measured by normalized jerk) peaked in late teens, that is, at the age of biological maturity. Ability to produce force (measured by peak acceleration) peaked later in life.^6^ Finally, measures of error in gross motor performance (e.g., movement errors) generally declined throughout adulthood, consistent with prior findings.^7^ Taken together, the clear relationships between age and performance found in our online data and that these relationships are consistent with existing knowledge provide compelling evidence of the validity of these baseline data.

### Summary Statistics for Clinical Participants

Participants with ataxia and parkinsonism differed from age‐matched online controls across a number of Hevelius movement features. In particular, features related to duration (movement time, execution time, number and duration of pauses, and click duration) were increased, and those related to movement control (distance from target at end of main submovement noise‐to‐force ratio, and jerk) were impaired compared with online controls in both ataxia and parkinsonism (see Supplementary Table S2).

Participants with ataxia demonstrated additional impairments in features reflecting “dysmetria”: direction changes, target reentries, movement error and variability, and deviation from task axis. Similarly, in participants with parkinsonism but not ataxia decreased peak acceleration and peak speed were present, matching the phenotype of “bradykinesia.” These observations are illustrated in Supplementary Figure S3 (bottom).

### Clinical Score Estimation

Table 2 shows the performance of regression models trained to predict clinical severity scores. For both ataxia and parkinsonism, we separately predicted dominant arm scores and total scores. We also introduced a disease‐independent “common score”: disease‐specific dominant arm and total scores were normalized by the maximum score to obtain a value between 0 and 1.

**Table:** 

Comparison (number in parentheses next to each class)	Number of features used	Sensitivity	Specificity	Positive predictive value	Negative predictive value
Parkinsonism (46) versus healthy (29)	5	0.913	1.000	1.000	0.879
Ataxia (95) versus healthy (29)	4	0.926	0.897	0.967	0.788
Mild ataxia (16) versus healthy (29)	6	0.750	0.966	0.923	0.875
Ataxia (95) versus parkinsonism (46)	10	0.853	0.913	0.953	0.750
Ataxia (68) versus parkinsonism (46), age ≥ 45	11	0.897	0.891	0.924	0.854
Ataxia (21) versus healthy (26), age ≤ 37	2	0.857	0.923	0.900	0.889

As described in the text, “mild ataxia” refers to the subset of participants who have a BARS dominant arm subscore of 0. Features selected by each classification model are shown in Supplementary Table S4.

**Table 2:** Results of the regression analyses (top) and classification analyses (bottom)

Clinical score estimated (score range in parentheses)	Number per diagnosis	Mean absolute error (MAE)	MAE as a percentage of maximum score	Correlation between clinical score and estimated score from regression models (*r*)
BARS dominant arm (0–4)	Ataxia, 91; controls, 29	0.35 ± 0.056	8.9% ± 1.4%	0.78, *P* < 0.0001
BARS total (0–30)	ataxia, 83; controls, 29	2.82 ± 0.582	9.4% ± 1.6%	0.83, *P* < 0.0001
UPDRS dominant arm total (0–24)	parkinsonism, 44; controls, 29	1.51 ± 0.283	6.3% ± 1.2%	0.66, *P* < 0.0001
UPDRS total (0–108)	parkinsonism, 44; controls, 29	5.80 ± 1.360	5.4% ± 1.3%	0.73, *P* < 0.0001
Common dominant arm (0–1)	ataxia, 91; parkinsonism, 44; controls, 29	0.09 ± 0.011	8.6% ± 1.1%	0.75, *P* < 0.0001
Common total (0–1)	ataxia, 83; parkinsonism, 44; controls, 29	0.08 ± 0.017	8.2% ± 1.7%	0.83, *P* < 0.0001

Severity scores were unavailable for a small number of patients (in addition, for some ataxia patients only dominant arm scores were available, but not total scores); hence, the number of participants included in the regression analyses differs from the number included in the classification analyses. Median 95% within‐session confidence intervals shown in columns 3 and 4 were estimated using the bootstrap method and reflect the sensitivity of score predictions to natural variability in performance during a single assessment session. Features selected by each regression model are shown in Supplementary Table S4.

The estimates produced by the regression models correlated strongly with actual clinical scores. The correlation coefficient ranged from *r* = 0.66 for UPDRS dominant arm total to *r* = 0.83 for Brief Ataxia Rating Scale (BARS) total and common total score. The mean absolute error (MAE) for all was <10% of the maximum score. The MAE for Hevelius ± standard deviation (SD) in estimating BARS dominant arm score was 0.35 ± 0.30, comparable to the previously published MAE of 0.38 of expert clinicians asked to rate video recordings of the finger‐nose‐finger task.^8^

Although Hevelius measures dominant arm performance, it is equally effective for predicting dominant arm score and total score. This is not surprising given that in our data set dominant arm score and total score were highly correlated (BARS, *r* = 0.89, *P* < 0.0001; UPDRS, *r* = 0.82, *P* < 0.0001; common score, *r* = 0.85, *P* < 0.0001).

The results of the bootstrap analysis indicated high within‐session reliability of the severity score estimates (Table 2).

### Classification Analyses

Classification models trained on data produced by Hevelius distinguished between individual disease classes (ataxia or parkinsonism) and healthy controls with high sensitivity (≥0.91) and specificity (≥0.90); see Table 2. As expected, different features were most informative for different phenotypes (see Supplementary Table S4). A model discriminating ataxia and parkinsonism patients also demonstrated good performance (sensitivity, 0.85; specificity, 0.91).

A model trained to discriminate between healthy controls and early‐stage ataxia patients (BARS score of 0 in the dominant arm), yielded a sensitivity of 0.75 and specificity of 0.97.

### Clinical Progression Estimation

A binary classification model trained to learn which session in a pair of sessions was more severe was applied to 18 individuals with a progressive ataxia diagnosis and a repeat session (12 with SCA, 4 with A‐T, and 2 with MSA‐C). The mean interval duration between sessions was 325 days with a range of 126–469 days. In these 18 individuals, the dominant arm BARS score increased (indicating disease progression) in 8 of 18, was unchanged in 9 of 18, and decreased (indicating improvement) in 1 of 18 (an individual with SCA‐6). The classification model predicted that 17 of 18 individuals had increased dominant arm severity at the time of their second session. One of 18 was predicted by the model to have decreased severity on the second session (the same individual with SCA‐6 who also showed improvement on BARS). These results support that Hevelius can sensitively capture arm severity progression information.

## Discussion

Hevelius is a novel tool for performing objective, granular, and rapid assessments of dominant arm motor function. We have demonstrated that the tool can be used in children and adults and forms an interpretable and multidimensional representation of ataxia and parkinsonism.

We have shown that the 32 movement features computed from computer mouse trajectories are interpretable, capture several dimensions of motor control, and vary with development and aging (Supplementary Fig. S3). Regression models used these features to accurately estimate disease scores in individuals with ataxia or parkinsonism (Table 2), and another machine‐learning model detected severity progression in 17 individuals with ataxia. Accuracy in estimating dominant arm score in ataxia participants was comparable to the accuracy of clinical experts. Furthermore, the tool was shown to have high intrasession reliability. Thus, Hevelius produces granular, accurate, reliable, and age‐normalized assessments of arm function in ataxia and parkinsonism and may prove useful in related disorders affecting motor control.

An ideal screening tool for detecting early disease would not only coarsely discriminate disease from healthy states, but would also have disease specificity. It was for this reason that we tested the ability of Hevelius to distinguish between ataxia and parkinsonism (which it performed accurately; Table 2). In addition, Hevelius was able to accurately classify healthy individuals from the subset of ataxia participants who had no scorable abnormalities in the dominant arm, with only 1 false‐positive (Table 2). Thus, this tool could form part of an early screening technology, especially if combined with tools in additional domains, such as eye movement and speech analysis.

Many technologies have been developed in the last decade and a half to enable objective assessments of motor performance of individuals with neurologic diseases. Most rely on accelerometers^9^, ^10^; however, other useful scalable approaches have included spiral drawing on a tablet^11^ and keyboard typing.^12^ Our approach complements prior work in important ways. First, a computer with a mouse is a highly accessible technology, more so than specialized wearable devices and even more so than smartphones, especially for adults aged 65 and older.^13^ Second, although accelerometers give access to acceleration, our approach directly measures the hand's position and speed. This turns out to be important: of the 8 features used to discriminate disease from controls, 4 relied on position and 2 on speed (see Supplementary Table S4).

Another key feature is that Hevelius is scalable: the task took patients 2–6 minutes to complete and only requires a computer, a mouse, and an Internet connection without the need for special software. The simplicity of the task and the automated scoring mean that no special expertise is needed to use Hevelius. Accessibility, along with a design that engaged intrinsic motivation (curiosity^14^ and social comparison^15^), facilitated the collection of data from 500,000 online volunteers in 4 months. This raises the possibility that Hevelius could be used in the future to perform longitudinal assessments from thousands of individuals with neurodegenerative disease in their home setting.

There are several limitations to the current study. First, the normative data were collected from a self‐selected sample of online volunteers. It is possible that people who have the means and the time to access the Internet for personal reasons have better than average access to health care and, consequently, are healthier than average. Second, the largely cross‐sectional design does not enable an assessment of learning effects with shorter time scales or influences because of changes in the testing environment. Last, there were substantial age differences in different populations studied (ataxia, parkinsonism, controls). Despite age adjustment enabled by the normative data set, it is conceivable that not all age‐related factors were fully removed, resulting in inflated performance estimates of classification models.

## Authors’ Roles

Krzysztof Z. Gajos, PhD, Harvard John A. Paulson School of Engineering and Applied Sciences, Cambridge, MA: conception, organization, execution of the research project; design, execution, and review and critique of the statistical analysis; 3. writing of the first draft and review and critique of the manuscript. Katharina Reinecke, PhD, Paul G. Allen School of Computer Science and Engineering, University of Washington, Seattle, WA: execution of research project; review and critique of manuscript. Mary Donovan, BS, Department of Neurology, Massachusetts General Hospital, Harvard Medical School, Boston, MA: execution of the research project. Christopher D. Stephen, MB, ChB, Ataxia and Movement Disorders Units, Department of Neurology, Massachusetts General Hospital, Harvard Medical School, Boston, MA: execution of the research project; review and critique of the manuscript. Albert Y. Hung, MD, PhD, Movement Disorders Unit, Department of Neurology, Massachusetts General Hospital, Harvard Medical School, Boston, MA: execution of the research project; review and critique of the manuscript. Jeremy D. Schmahmann, MD, Ataxia Unit, Department of Neurology, Massachusetts General Hospital, Harvard Medical School, Boston, MA: execution of the research project; 3. review and critique of the manuscript. Anoopum S. Gupta, MD, PhD, Ataxia and Movement Disorders Units, Department of Neurology, Massachusetts General Hospital, Harvard Medical School, Boston, MA:. conception, organization, and execution of the research project; review and critique of the statistical analysis; writing of the first draft and review and critique of the manuscript.

## Authors' Full Financial Disclosures

Krzysztof Z. Gajos, PhD, is employed by Harvard University and has received grants from NIH, NSF, and Adobe. Katharina Reinecke, PhD, is employed by the University of Washington, has received grants from NSF CAREER Grant, and gift money from Adobe Research, Microsoft, and Google CSO of Startup Augury Design Inc. Mary Donovan, BS, is employed by Massachusetts General Hospital and is a medical student at Georgetown University. Christopher D. Stephen, MB, ChB, is employ8ed by Massachusetts General Hospital, has received grants from Sanofi‐Genzyme, has receceived compensation for conducting clinical trials from Sanofi‐Genzyme, Bristol‐Myers Squibb, Biogen Inc., and Biohaven Pharmaceuticals Inc. Albert Y. Hung, MD, PhD, is employed by Massachusetts General Hospital. Jeremy D. Schmahmann, MD, has stock ownership Cadent Pharmaceuticals, intellectual property rights in the Brief Ataxia Rating Scale, Cerebellar Cognitive Affective Syndrome Scale, and Cerebellar Neuropsychiatric Rating Scale and is a license holder with the General Hospital Corporation, is a consultant for Cadent and Biohaven, has given expert testimony for the Massachusetts Committee for Public Counsel Services. is on advisory boards of Cadent, National Ataxia Foundation, Society for Research on Cerebellum and Ataxias, is employed by Massachusetts General Hospital, has received honoraria from the Institute of Neurology Queen Square, British Neuropsychiatric Association, Spanish Neurological Society, New York University Langone Medical Center, American Speech‐Language‐Hearing Association, Department of Neurology Emory University, Montefiore Medical Center and Albert Einstein School of Medicine, has received royalties from Oxford University Press, Elsevier, Springer, MacKeith Press, has received grants from National Ataxia Foundation, NIH, US Army Medical Research, and has received compensation for conducting clinical trials and sponsored research from Biohaven Pharmaceuticals Inc. Anoopum S. Gupta, MD, PhD, was a consultant for Biogen, Inc., is employed by Massachusetts General Hospital, has received grants from Ataxia‐Telangiectasia Children's Project, Biogen, Inc.

## Supporting information


**Appendix S1**: Supplementary Methods
