# Supplementary material for: Computer mouse use captures ataxia and parkinsonism, enabling accurate measurement and detection
Source: Mov Disord. 2019 Nov 7;35(2):354–8. doi: 10.1002/mds.27915 (PMC7028247; doi:10.1002/mds.27915)
Supplement: Supplementary file 1 — Appendix S1: Supplementary Methods [file MDS-35-354-s001.docx]

# Supplementary Methods

## Standard protocol approvals, registrations, and patient consents

## The online collection of the normative data was approved by the Harvard University Internal Review Board. Participants were presented with an informed consent form online and provided consent by clicking the “I agree” button.

## For the clinical data collection, consent/assent and protocols were approved by the Partners Healthcare Institutional Review Board.

## Overall design of the Hevelius tool

Hevelius is a browser-based software tool for quantifying a person’s motor performance in the arm. Hevelius builds on a large body of prior research that used quantitative measures derived from two-dimensional movement trajectories to characterize aspects of the development, aging and abnormality of the human neuro-motor system [^1–10^](https://paperpile.com/c/lboMzj/WkMYm+newgB+9vdOV+wbovG+H7puV+kudAa+MVE3a+MB5LY+k1OEU+iyy19). Hevelius presents participants with a set of pointing tasks to be performed with a computer mouse, collects detailed data about each movement, and computes 32 measures (table S1) that quantify several aspects of a participant’s performance. The measurements are then compared to the baseline data collected from healthy volunteers of the same age and are reported as age-specific z-scores.

## Tasks

Participants were instructed to use the mouse to click on a target (a red circle) on a computer screen as soon as it appeared. One target was presented at a time and as each target was correctly clicked, the next target appeared. Clicking on a single target constituted a complete trial. A sequence of nine targets constituted a block. Participants were allowed to rest between blocks, but were instructed to complete each block without interruption.

Participants were presented with 10 blocks of 9 targets each. During in-clinic deployment, the first two blocks were for practice and were excluded from analyses.

The first pointing task in each block involved having the participant position the mouse cursor in a known position, while the remaining 8 tasks were used for the actual assessment.

The blocks differed in terms of the sizes of the targets and distances between successive targets to cover the range of tasks typically experienced when operating a computer and to improve the robustness of the measurements. During in-clinic deployment, target sizes were varied from 20 to 60 pixels and distances between targets were varied from 150 to 800 pixels such that the index of difficulty (computed as [
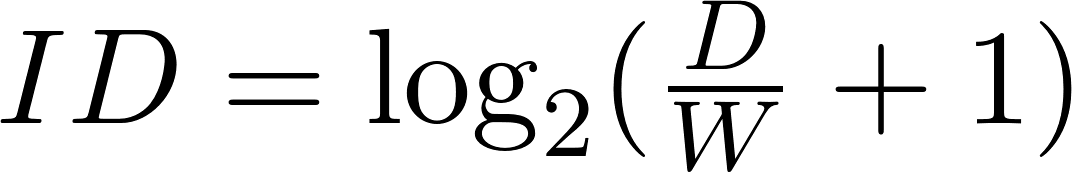
](https://www.codecogs.com/eqnedit.php?latex=ID%3D%5Clog_2%5C(%5Cfrac%7BD%7D%7BW%7D%2B1%5C)%250), where D is the distance to the next target and W is the target diameter) ranged from 2.2 to 4.8. The properties of individual tasks and their order were fixed across all participants. During online deployment, target sizes varied from 15 to 60 pixels. Distances between targets varied similarly to the clinical deployment, but if a participant used a device with a small screen, the distances were automatically adapted such that the task would fit on the screen.

## Processing of movement data and feature extraction

The raw data collected included basic movement statistics (location of endpoints, timing) as well as detailed movement trajectories. The movement trajectories were translated, rotated, resampled, and smoothed. Because discrete sampling of continuous mouse pointer trajectories introduces potential artifacts (such as discontinuities in direction and speed, which would produce large noise in derivatives), we adopted an approach similar to the one previously published[^7,23^](https://paperpile.com/c/lboMzj/d0V6e+MVE3a) to translate, rotate, resample and smooth the data prior to computing any measures. Specifically, we first translated and rotated the movement trajectories such that each movement started at the origin and ended on the x-axis. We then resampled movement trajectories at a 100Hz rate. Finally, movement trajectories were smoothed using a Kalman filter. We computed speed using the discrete derivative of the smoothed 2D pointer positions with respect to time and the result was smoothed using a 7Hz low-pass FIR filter (with 40dB stopband attenuation using Kaiser window). To compute acceleration and jerk, we similarly first computed discrete derivatives of speed and acceleration, respectively, and applied the same low-pass filter.

Following prior work[^7,11^](https://paperpile.com/c/lboMzj/MVE3a+Y70Ue) (and as illustrated in figure S1), we decomposed each movement into several components: initiation time (from the target onset to the first mouse move event), execution time (from first to last mouse move event), verification time (time spent inside the target between last mouse move event and the start of the click), and click (time from mouse down to mouse up event). We marked pauses whenever there was a break of 100 ms or more in the raw mouse movement events.

A new submovement was marked when the speed crossed the 100 pixels/s threshold, but only if it subsequently reached at least 500 pixels/s (see figure S2). The end of the movement was marked when the speed fell again below 100 pixels/s. Three of the measures reported by Hevelius required the identification of the main submovement. The main submovement was identified as the submovement during which the speed reached its maximum value.

## Computing age-specific z-scores

Age-specific z-scores for the in-clinic participants were computed by comparing an individual’s results to those of healthy online volunteers of exactly the same age. The overall z-scores were smoothed across neighboring ages using a locally-weighted linear regression (with [
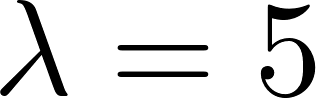
](https://www.codecogs.com/eqnedit.php?latex=%5Clambda%20%3D%205%250))[^12^](https://paperpile.com/c/lboMzj/dVZLT). The z-scores were computed separately for each block of trials and later averaged across blocks.

### Computing age-specific normative baselines

Pointing performance varies with the size of the target and the distances between targets[^24^](https://paperpile.com/c/lboMzj/ok8lD). To allow our baseline values to be independent of the task properties, we performed the following steps:

1. We averaged values of each measure per block.
2. We applied the Box-Cox transform[^25,26^](https://paperpile.com/c/lboMzj/wj3nj+TMxor) to make the distribution of the values of each measure approximately normal.
3. For each measure, we fitted a regression of the form: [
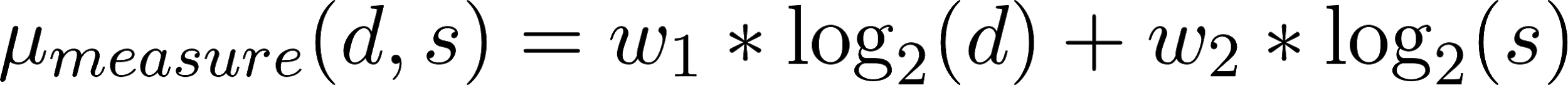
](https://www.codecogs.com/eqnedit.php?latex=%5Cmu_%7Bmeasure%7D(d%2Cs)%20%3D%20w_1%20*%20%5Clog_2(d)%20%2B%20w_2%20*%20%5Clog_2(s)%250),
   Where *d* is the distance between successive targets, *s* is the target size, and [
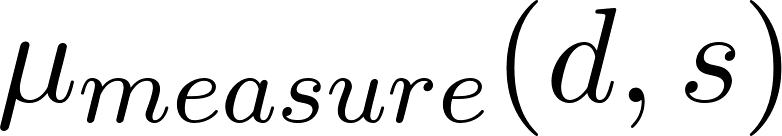
](https://www.codecogs.com/eqnedit.php?latex=%5Cmu_%7Bmeasure%7D(d%2Cs)%250) is the Box-Coxed transformed value of a measure.
4. For each measure and each age, we computed the standard deviation of the residuals.

Z-scores for future test-takers were computed by computing the difference between the test-taker’s performance and the performance estimated by [
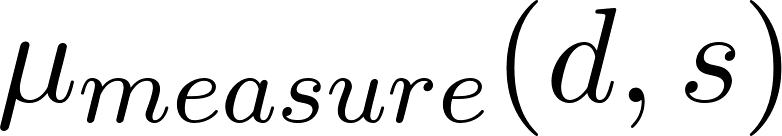
](https://www.codecogs.com/eqnedit.php?latex=%5Cmu_%7Bmeasure%7D(d%2Cs)%250) and dividing the difference by the standard deviation of the residuals.

## Collection and processing of normative data online

To collect large quantities of normative data from diverse participants, we launched a study on LabintheWild.org[^13^](https://paperpile.com/c/lboMzj/zn5CR), a platform for conducting large-scale behavioral research with unpaid online volunteers.

### Participant selection

Most participants arrived at the study site via search, social media referrals or other referrals. All were allowed to participate, but only a subset were included in the final analyses: those who reported using a mouse with their dominant hand and who revealed their age. 3.9% of otherwise eligible participants were excluded because they answered “yes” to the question “Do you have any medical condition that might affect how you use a computer?” Analysis of a random sample of 100 open-ended explanations showed that the most common conditions reported were vision impairments (n=30), carpal tunnel (n=13), ADHD (n=6), arthritis (n=6), multiple conditions (n=5), color vision deficiency (n=4), multiple sclerosis (n=3), and use of non-dominant hand (n=2). In addition, we excluded participants aged ≥86 and ≤4, given low sample sizes at these ages.

### Online data collection procedures

Collecting data from unpaid online volunteers has the advantage that there is no registration necessary for reimbursement, which could impose a sign-up barrier and deter certain populations[^14,15^](https://paperpile.com/c/lboMzj/Z0Sw1+LaIPH). We followed an existing approach and best practices for obtaining valid data from uncompensated online volunteers[^13,16^](https://paperpile.com/c/lboMzj/zn5CR+Uz7wU): we incentivized participation by providing personalized feedback on their performance at the end of the study and provided opportunities for participants to flag situations that could have compromised data (participants were asked if they had taken the study before, if they experienced any technical difficulties, were interrupted during the study or provided any false information). Research has shown that studies that follow these practices produce data that are comparable to those obtained in conventional laboratory studies and accurately replicate in-lab study results[^13,16–18^](https://paperpile.com/c/lboMzj/Uz7wU+10NLe+zn5CR+CyDyO).

## Collection of clinical data

### Participant selection

Participants were recruited from the Massachusetts General Hospital (MGH) between August 2016 and July 2018. Individuals in the Ataxia and Movement Disorders Units, regardless of specific diagnosis, were invited to participate if they reported being able to use a computer mouse. Additionally, children with ataxia-telangiectasia (A-T) were recruited through the Ataxia-Telangiectasia Children’s Project or the MGH Ataxia Unit. Individuals were invited (but not required) to repeat a testing session at a subsequent visit to MGH. Healthy subject data were obtained from two populations: 1) family members of patients (e.g., asymptomatic partners or gene negative family members) and 2) MGH clinical and research staff. Participants used Hevelius with their dominant arm (e.g., individuals who identified themselves as right-handed were asked to perform the task with their right arm). In cases of handedness ambiguity, individuals were asked to use the arm that they would typically use a computer mouse with or write with. Clinical data for MGH patients including diagnosis and scores on clinical rating scale were identified in the medical record from their concurrent visit. All patients had disease-specific rating scale scores and for those without a same-day clinical appointment, scores were obtained from video data of the same-day neurological exam.

### Clinical data collection procedures

All neurologic examinations were videotaped. Ataxia patients were scored on the Brief Ataxia Rating Scale (BARS) (range 0–30). Patients with parkinsonism were assessed with the Unified Parkinson’s Disease Rating Scale (UPDRS) Part III Motor Examination (range 0–108)[^19^](https://paperpile.com/c/lboMzj/Fg7DO). For regression models combining patients with ataxia and parkinsonism, dominant arm and total scores were normalized by the maximum score to obtain a value between zero and one and allow comparison between patients with different diseases. Whereas the BARS has a single score for each arm (range 0–4) based on the finger-nose-finger task, the UPDRS has several scoring elements for each arm (rest tremor (0–4), postural tremor (0–4), rigidity (0–4), and bradykinesia on three tasks (0–12)), which were summed to form a composite severity score for the dominant arm (0–24).

## Clinical Score Estimation

To estimate clinical scores from Hevelius measurements, we used least absolute shrinkage and selection operator (LASSO)[^20^](https://paperpile.com/c/lboMzj/huqPT), a regression method that simultaneously performs feature selection and fits a linear model. We used 20-fold cross-validation to evaluate the predictive performance of the regression models using mean absolute error (MAE) and the Pearson correlation coefficients as quantitative measures of model performance. Bootstrap validation[^21^](https://paperpile.com/c/lboMzj/yBEet) was used to estimate the sensitivity of score predictions to variability in performance during a single assessment session. We produced bootstrap samples by resampling (with replacement) 8 blocks, recomputing the mean values of each measure for each participant, and computing new regression values for each sample. For each analysis, we computed 1000 bootstrap samples.

## Classification Analysis

We used a linear support vector machine (SVM) with the regularization parameter C=1 for classification. Because Hevelius produces several correlated measures, we first applied a feature selection algorithm (recursive feature elimination[**^22^**](https://paperpile.com/c/lboMzj/oZDtn) followed by a variant of the stepwise discriminant process) to identify a subset of features that were most informative for any given classification task. We used leave-one-out cross-validation to estimate the performance of the classifiers. All classifiers were trained and evaluated using age-specific z-scored features (see section above) in order to account for age differences present across groups.

## Disease Progression Analysis

We trained a logistic regression classification model (with L1 regularization) to identify which individual in a pair had more severe disease. The input to the model was 1) the numeric difference in the 32 Hevelius features between two individuals (individual A minus individual B) and 2) the binary variable indicating which individual’s dominant arm score (on BARS or UPDRS) was higher. If all pairwise comparisons between $N$participants were considered, there would be $N*(N-1)/2$unique comparisons. However, comparisons between individuals with the same score were excluded and individuals with ataxia were only compared with other individuals with ataxia or controls (similarly for parkinsonism). Information about which individual in each pair was more severe was used by the model to then predict whether the first or second session from a single individual was more severe. 18-fold cross-validation was used to evaluate the performance of this classifier. In each fold, data from one of the 18 ataxia patients with repeat visits was held out for evaluation. This enabled us to determine, for individuals with a progressive ataxia diagnosis (SCA, A-T, or MSA-C), how often the model estimated more severe disease at the second time point. Repeat sessions from individuals with other diagnoses (e.g., Parkinson’s disease) were not included in this analysis as assessments of progression would be confounded by symptomatic treatment.

## Implementation

All machine learning and numerical analyses were implemented in Python 3.6.2 with the following key libraries: numpy 1.13.1, pandas 0.20.3, scikit-learn 0.19.0 or in Mathworks MATLAB.

# Supplementary Results

## Classification analyses

To assess whether age-specific z-scored features improved the performance of our models, we performed additional ataxia versus parkinsonism and ataxia versus healthy classification using age-specific z-scored and age-generic z-scored features. As shown in [table **S3**](https://docs.google.com/document/d/1K8VOUHkE6cJGaQzppAadOowjay1SxcoIDG9k5K7Mukg/edit#tab_classification), performance for ataxia versus parkinsonism improved substantially with age-specific z-scores. For ataxia versus healthy classification, overall performance was largely unchanged, likely due to the fact that the children with A-T tended to have moderate to severe disease, such that classification was not impacted by age-specific z-scoring (one would expect more benefit for populations with mild disease). As expected, the age-specific z-scored model showed higher specificity (but lower sensitivity), consistent with the finding that accounting for age in children resulted in fewer false positives (i.e., fewer healthy children labeled as having ataxia).

## References

1. [Keates, S. & Trewin, S. Effect of age and Parkinson’s disease on cursor positioning using a mouse. in *Assets ’05: Proceedings of the 7th international ACM SIGACCESS conference on Computers and accessibility* 68–75 (ACM Press, 2005). doi:](http://paperpile.com/b/lboMzj/WkMYm)[10.1145/1090785.1090800](http://dx.doi.org/10.1145/1090785.1090800)

2. [Keates, S., Hwang, F., Langdon, P., Clarkson, P. J. & Robinson, P. Cursor measures for motion-impaired computer users. in *Assets ’02: Proceedings of the fifth international ACM conference on Assistive technologies* 135–142 (ACM, 2002). doi:](http://paperpile.com/b/lboMzj/newgB)[http://doi.acm.org.ezp-prod1.hul.harvard.edu/10.1145/638249.638274](http://dx.doi.org/http://doi.acm.org.ezp-prod1.hul.harvard.edu/10.1145/638249.638274)

3. [Hurst, A., Hudson, S. E., Mankoff, J. & Trewin, S. Automatically detecting pointing performance. in *IUI ’08: Proceedings of the 13th international conference on Intelligent user interfaces* (ACM Press, 2008).](http://paperpile.com/b/lboMzj/9vdOV)

4. [Hourcade, J. P., Bederson, B. B., Druin, A. & Guimbretière, F. Differences in pointing task performance between preschool children and adults using mice. *ACM Trans. Comput. -Hum. Interact.* **11**, 357–386 (2004).](http://paperpile.com/b/lboMzj/wbovG)

5. [Donker, A. & Reitsma, P. Aiming and clicking in young children’s use of the computer mouse. *Comput. Human Behav.* **23**, 2863–2874 (2007).](http://paperpile.com/b/lboMzj/H7puV)

6. [Cooke, J. D., Brown, S. H. & Cunningham, D. A. Kinematics of arm movements in elderly humans. *Neurobiol. Aging* **10**, 159–165 (1989).](http://paperpile.com/b/lboMzj/kudAa)

7. [Walker, N., Philbin, D. A. & Fisk, A. D. Age-related differences in movement control: adjusting submovement structure to optimize performance. *J. Gerontol. B Psychol. Sci. Soc. Sci.* **52B**, P40–52 (1997).](http://paperpile.com/b/lboMzj/MVE3a)

8. [Walker, N., Meyer, D. E. & Smelcer, J. B. Spatial and temporal characteristics of rapid cursor-positioning movements with electromechanical mice in human-computer interaction. *Hum. Factors* **35**, 431–458 (1993).](http://paperpile.com/b/lboMzj/MB5LY)

9. [Cheong, Y., Shehab, R. L. & Ling, C. Effects of age and psychomotor ability on kinematics of mouse-mediated aiming movement. *Ergonomics* **56**, 1006–1020 (2013).](http://paperpile.com/b/lboMzj/k1OEU)

10. [Ketcham, C. J., Seidler, R. D., Van Gemmert, A. W. A. & Stelmach, G. E. Age-related kinematic differences as influenced by task difficulty, target size, and movement amplitude. *J. Gerontol. B Psychol. Sci. Soc. Sci.* **57**, P54–64 (2002).](http://paperpile.com/b/lboMzj/iyy19)

11. [Wobbrock, J. O. & Gajos, K. Z. Goal Crossing with Mice and Trackballs for People with Motor Impairments: Performance, Submovements, and Design Directions. *ACM Trans. Access. Comput.* **1**, 1–37 (2008).](http://paperpile.com/b/lboMzj/Y70Ue)

12. [Hastie, T., Tibshirani, R. & Friedman, J. *The elements of statistical learning: data mining, inference and prediction*. (Springer, 2009).](http://paperpile.com/b/lboMzj/dVZLT)

13. [Reinecke, K. & Gajos, K. Z. LabintheWild: Conducting Large-Scale Online Experiments With Uncompensated Samples. in *Proceedings of the 18th ACM Conference on Computer Supported Cooperative Work & Social Computing* 1364–1378 (ACM, 2015). doi:](http://paperpile.com/b/lboMzj/zn5CR)[10.1145/2675133.2675246](http://dx.doi.org/10.1145/2675133.2675246)

14. [Brewer, R., Morris, M. R. & Piper, A. M. ‘Why Would Anybody Do This?’: Understanding Older Adults’ Motivations and Challenges in Crowd Work. in *Proceedings of the 2016 CHI Conference on Human Factors in Computing Systems* 2246–2257 (ACM, 2016). doi:](http://paperpile.com/b/lboMzj/Z0Sw1)[10.1145/2858036.2858198](http://dx.doi.org/10.1145/2858036.2858198)

15. [Zyskowski, K., Morris, M. R., Bigham, J. P., Gray, M. L. & Kane, S. K. Accessible crowdwork?: Understanding the value in and challenge of microtask employment for people with disabilities. in *Proceedings of the 18th ACM Conference on Computer Supported Cooperative Work & Social Computing* 1682–1693 (ACM, 2015).](http://paperpile.com/b/lboMzj/LaIPH)

16. [Germine, L. *et al.* Is the Web as good as the lab? Comparable performance from Web and lab in cognitive/perceptual experiments. *Psychon. Bull. Rev.* **19**, 847–857 (2012).](http://paperpile.com/b/lboMzj/Uz7wU)

17. [Gosling, S. D., Vazire, S., Srivastava, S. & John, O. P. Should we trust web-based studies? A comparative analysis of six preconceptions about Internet questionnaires. *Am. Psychol.* **59**, 93–104 (2004).](http://paperpile.com/b/lboMzj/10NLe)

18. [Li, Q., Gajos, K. Z. & Reinecke, K. Volunteer-Based Online Studies With Older Adults and People with Disabilities. in *Proceedings of the 20th International ACM SIGACCESS Conference on Computers & Accessibility (ACM ASSETS 2018)* (2018).](http://paperpile.com/b/lboMzj/CyDyO)

19. [Fahn, S. & Elton, R. L. Unified Parkinson’s disease rating scale. *In: Fahn, S., Marsden, C.D., Calne, D. and Goldstein, M., Eds., Recent Developments in Parkinson’s Disease, Macmillan Health Care Information, Florham Park* 153–163 (1987).](http://paperpile.com/b/lboMzj/Fg7DO)

20. [Tibshirani, R. Regression shrinkage and selection via the lasso: a retrospective. *J. R. Stat. Soc. Series B Stat. Methodol.* **73**, 273–282 (2011).](http://paperpile.com/b/lboMzj/huqPT)

21. [Wasserman, L. *All of Statistics: A Concise Course in Statistical Inference*. (Springer Publishing Company, Incorporated, 2010).](http://paperpile.com/b/lboMzj/yBEet)

22. [Guyon, I., Weston, J., Barnhill, S. & Vapnik, V. Gene Selection for Cancer Classification using Support Vector Machines. *Mach. Learn.* **46**, 389–422 (2002).](http://paperpile.com/b/lboMzj/oZDtn)

23. [Gajos, K., Reinecke, K. & Herrmann, C. Accurate measurements of pointing performance from in situ observations. in *Proceedings of the 2012 ACM annual conference on Human Factors in Computing Systems* 3157–3166 (ACM, 2012). doi:](http://paperpile.com/b/lboMzj/d0V6e)[10.1145/2208636.2208733](http://dx.doi.org/10.1145/2208636.2208733)

24. [Fitts, P. M. & Peterson, J. R. Information Capacity of Discrete Motor Responses. *J. Exp. Psychol.* **67**, 103–112 (1964).](http://paperpile.com/b/lboMzj/ok8lD)

25. [Box, G. E. P. & Cox, D. R. An analysis of transformations. *J. R. Stat. Soc. Series B Stat. Methodol.* **26**, 211–252 (1964).](http://paperpile.com/b/lboMzj/wj3nj)

26. [Sakia, R. M. The Box-Cox transformation technique: a review. *Statistician* 169–178 (1992).](http://paperpile.com/b/lboMzj/TMxor)

27. [MacKenzie, I. S., Kauppinen, T. & Silfverberg, M. Accuracy measures for evaluating computer pointing devices. in *Proceedings of the SIGCHI conference on Human factors in computing systems* 9–16 (ACM, 2001). doi:](http://paperpile.com/b/lboMzj/Mx4PA)[10.1145/365024.365028](http://dx.doi.org/10.1145/365024.365028)

28. [Balasubramanian, S., Melendez-Calderon, A. & Burdet, E. A robust and sensitive metric for quantifying movement smoothness. *IEEE Transactions on Biomedical Engineering* **59**, 2126–2136 (2012).](http://paperpile.com/b/lboMzj/ZGXYA)

29. [Hogan, N. & Sternad, D. Sensitivity of smoothness measures to movement duration, amplitude, and arrests. *J. Mot. Behav.* **41**, 529–534 (2009).](http://paperpile.com/b/lboMzj/vYu5m)

# Figure legends and tables

[**Figure S1**](#26in1rg). Components of a movement.


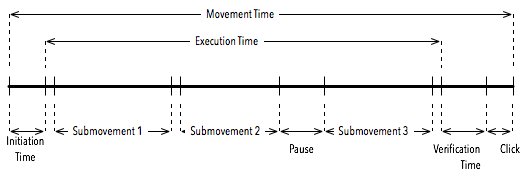


[**Figure S2**](#lnxbz9). We used speed thresholds to identify submovements.


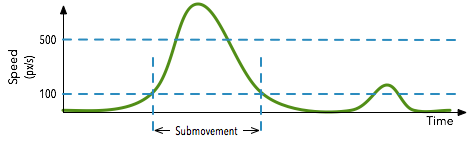


**[Figure](https://docs.google.com/document/d/1K8VOUHkE6cJGaQzppAadOowjay1SxcoIDG9k5K7Mukg/edit#figur_online_summary) S3.** Representative summaries of data collected. Top: Cross-sectional statistics depicting four measures as a function of age for online participants. Error bars indicate 95% confidence intervals. Bottom: Illustration of movement data from three in-clinic participants for 16 movements. The participants were aged 54, 59 and 60 (left to right). The ataxia (MSA-C) and PD patients had similar common dominant arm scores (0.375 for the ataxia patient and 0.33 for the PD patient). Gray lines show individual movements while the red lines show averages. Top row shows actual movement trajectories rotated and scaled such that all start at the origin and end at (1,0). Bottom two rows show speed and acceleration profiles with the x-axis representing normalized time (0=time of the start of the movement, 1=time of the successful click on the target).


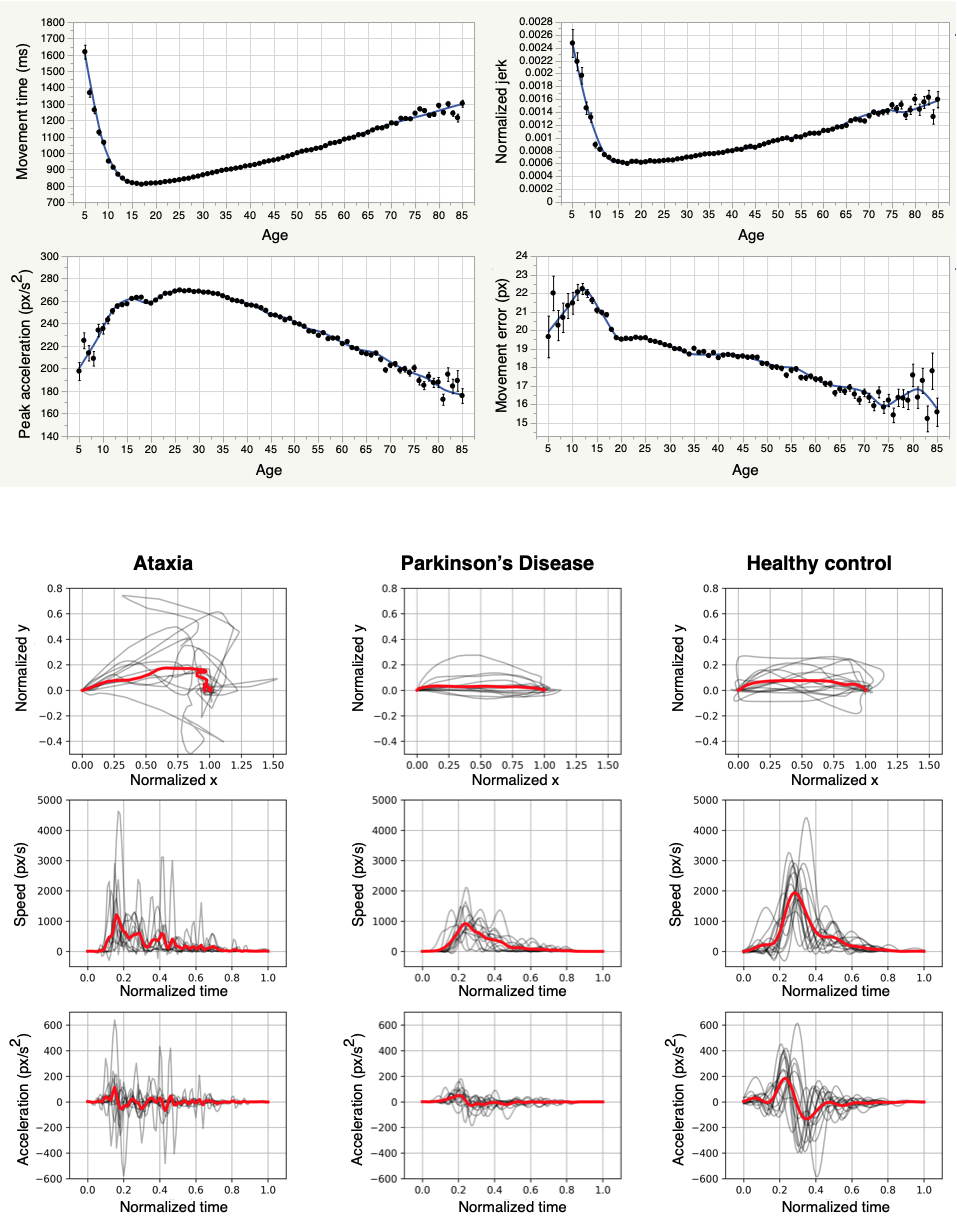


[**Table S1**](#35nkun2). Movement measures reported by Hevelius.

| **Movement time** | Complete movement time from target onset to the end of the successful click on the target. |
| --- | --- |
| **Movement time variability** | Coefficient of variation of movement times in a block of trials. |
| **Execution time** | Time from the first to the last mouse movement (excluding any movement that occurred while the mouse button was pressed -- see Click slip). |
| **Execution time without pauses** | Like execution time, but excludes pauses of 100ms or longer. |
| **Execution time variability** | Coefficient of variation of execution times in a block of trials. |
| **Execution time variability (without pauses)** | Coefficient of variation of execution times (without pauses) in a block of trials. |
| **Peak speed** | The maximum (smoothed) speed recorded during a movement. |
| **Peak speed variability** | Coefficient of variation of peak speeds in a block of trials. |
| **Peak acceleration** | The maximum (smoothed) acceleration recorded during a movement. |
| **Peak acceleration variability** | Coefficient of variation of peak accelerations in a block of trials. |
| **Distance from target center at end of main submovement** | The 2D distance from the mouse pointer location at the end of the main submovement to the target center. |
| **Fraction of remaining distance to the target center covered in main submovement** | The fraction of the remaining distance along the task axis covered during the main submovement. The value of this measure can be greater than 1 if the person overshoots the target. |
| **Maximum deviation from task axis** | The maximum distance of the mouse pointer from the task axis during a movement. |
| **Movement variability**[^27^](https://paperpile.com/c/lboMzj/Mx4PA) | The standard deviation of the distance of the actual path from the task axis. |
| **Movement error**[^27^](https://paperpile.com/c/lboMzj/Mx4PA) | The average absolute distance of the mouse pointer from the task axis. In other words, this measure captures, at the gross level, how far the pointer trajectory was from a straight line. |
| **Movement offset**[^27^](https://paperpile.com/c/lboMzj/Mx4PA) | The average (non-absolute) distance of the mouse pointer from the task axis. A large magnitude of movement offset indicates that the movement trajectory falls mostly to one side of the task axis or the other. A movement with a large movement error may still have a small movement offset if the path of the movement deviates first to one side of the movement axis and then to the other. |
| **Task axis crossings**[^27^](https://paperpile.com/c/lboMzj/Mx4PA) | The number of times the mouse pointer crossed the task axis during the movement. |
| **Target re-entries**[^27^](https://paperpile.com/c/lboMzj/Mx4PA) | The number of times the mouse pointer leaves the target and then re-enters it before the start of the click. |
| **Movement direction changes**[^27^](https://paperpile.com/c/lboMzj/Mx4PA) | The number of times the movement component orthogonal to the task axis changes sign. |
| **Orthogonal direction changes**[^27^](https://paperpile.com/c/lboMzj/Mx4PA) | The number of times the movement component parallel to the task axis changes sign. |
| **Main submovement** | The submovement with the highest peak speed. |
| **Verification time** | The time interval between the end of a movement inside a target and the beginning of the click (i.e., the time when the mouse button was pressed). |
| **Verification time variability** | Standard deviation of verification times in a block of trials. |
| **Click duration** | The time between mouse button press and release during the correct click on the target. |
| **Click duration variability** | Standard deviation of click durations in a block of trials. |
| **Click slip** | Distance between the point where the mouse button was pressed down and where it was released during click on the target. |
| **Noise-to-force ratio**[^7^](https://paperpile.com/c/lboMzj/MVE3a) | The standard deviation (computed over all trials in a block) of the distance from the target center at the end of the first submovement divided by mean of peak accelerations. |
| **Normalized jerk**[^28,29^](https://paperpile.com/c/lboMzj/ZGXYA+vYu5m) | A dimensionless measure computed as  [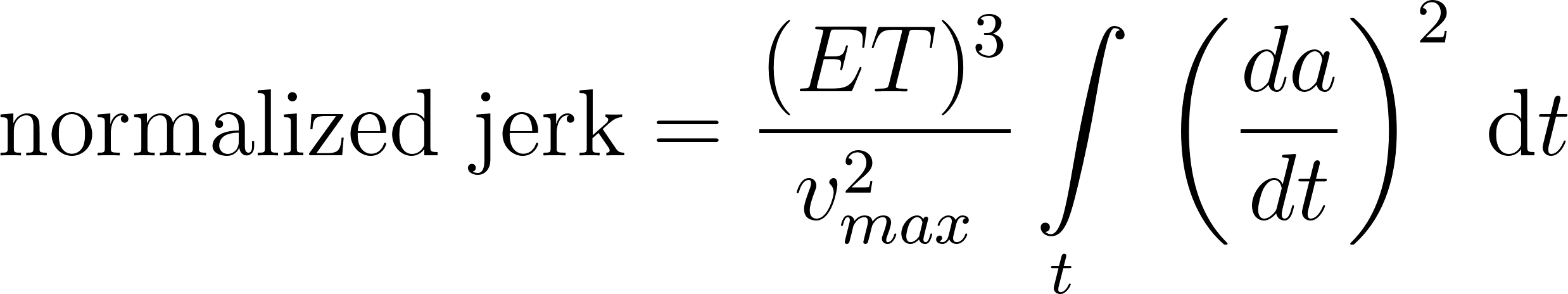](https://www.codecogs.com/eqnedit.php?latex=%5Cmbox%7Bnormalized%20jerk%7D%20%3D%20%5Cfrac%7B(ET)%5E3%7D%7Bv_%7Bmax%7D%5E2%7D%20%5Cint%5Climits_t%20%5C%2C%20%5Cleft(%5Cfrac%7Bda%7D%7Bdt%7D%5Cright)%5E2%20%5C%2C%20%5Cmbox%7Bd%7D%20t%20%251)  where [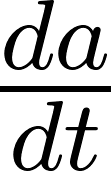](https://www.codecogs.com/eqnedit.php?latex=%5Cfrac%7Bda%7D%7Bdt%7D%250) is the jerk, [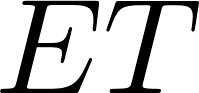](https://www.codecogs.com/eqnedit.php?latex=ET%250) is the execution time without pauses and [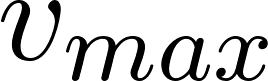](https://www.codecogs.com/eqnedit.php?latex=v_%7Bmax%7D%250) is the peak speed during the movement. |
| **Normalized jerk without pauses** | Like normalized jerk, but excludes parts of the movement when the mouse pointer was paused for 100ms or longer. |
| **Fraction of the main submovement spent accelerating** | The fraction of the time from the start of the submovement to the time when acceleration reached its peak value divided by the overall duration of the submovement. |
| **Number of pauses** | Number of pauses of 100ms or longer. |
| **Duration of the longest pause** | Duration of the longest pause of 100ms or longer. If not such pause occurred, 0ms is recorded for this measure. |

In several definitions, we refer to a task axis, which is the straight line linking the positions of the cursor at the start and at the end of the movement.

**[Table S2](#1ksv4uv)**. Summary statistics for patients with parkinsonism and ataxia

| **Measure** | **parkinsonism z-scores**  **mean (stdev)** | | | **ataxia z-scores**  **mean (stdev)** | | | **mild ataxia z-scores**  **mean (stdev)** | | | **P-value for difference between parkinsonism and ataxia** |
| --- | --- | --- | --- | --- | --- | --- | --- | --- | --- | --- |
| **Noise to force ratio** | **1.07** | (0.86) | **** | 0.92 | (0.85) | **** | 0.58 | (0.84) | * | 0.3264 |
| **Peak acceleration variability** | 0.54 | (0.59) | **** | 0.95 | (0.59) | **** | 0.61 | (0.57) | *** | 0.0003 |
| **Peak speed variability** | 0.61 | (0.56) | **** | **1.24** | (0.67) | **** | 0.81 | (0.6) | **** | 0.0000 |
| **Click duration variability** | 0.74 | (1.15) | **** | **1.17** | (1.23) | **** | 0.26 | (0.66) |  | 0.0414 |
| **Verification time variability** | 0.77 | (0.83) | **** | **1.60** | (1.08) | **** | 0.84 | (0.97) | ** | 0.0000 |
| **Movement time variability** | 0.35 | (0.52) | **** | 0.86 | (0.67) | **** | 0.36 | (0.46) | ** | 0.0000 |
| **Execution time variability** | 0.36 | (0.51) | **** | 0.75 | (0.59) | **** | 0.35 | (0.5) | * | 0.0001 |
| **Execution time (w/o pauses) variability** | 0.14 | (0.42) | * | 0.59 | (0.6) | **** | 0.03 | (0.28) |  | 0.0000 |
| **Movement time** | **1.87** | (1.2) | **** | **3.29** | (1.52) | **** | **1.63** | (1.27) | *** | 0.0000 |
| **Peak acceleration** | -0.82 | (1.16) | **** | 0.46 | (1.4) | ** | 0.22 | (1.01) |  | 0.0000 |
| **Click duration** | **1.06** | (1.52) | **** | **1.95** | (1.75) | **** | 0.33 | (1.16) |  | 0.0023 |
| **Click slip** | 0.09 | (0.86) |  | 0.33 | (0.84) | *** | 0.03 | (0.77) |  | 0.1231 |
| **Distance from target center at the end of the main submovement** | 0.81 | (0.69) | **** | **1.65** | (0.94) | **** | 0.88 | (0.62) | **** | 0.0000 |
| **Execution time** | **1.78** | (1.45) | **** | **3.77** | (2.28) | **** | **1.60** | (1.46) | *** | 0.0000 |
| **Execution time (w/o pauses)** | **1.80** | (1.38) | **** | **3.57** | (2.31) | **** | **1.56** | (1.67) | ** | 0.0000 |
| **Fraction of the distance to the target center covered in main submovement** | -0.43 | (1.36) | * | **1.71** | (2.74) | **** | 0.12 | (1.2) |  | 0.0000 |
| **Fraction of the main submovement spent accelerating** | 0.41 | (0.65) | *** | 0.39 | (0.67) | **** | 0.09 | (0.77) |  | 0.8851 |
| **Max deviation from task axis** | -0.08 | (0.8) |  | **1.12** | (0.97) | **** | 0.61 | (0.54) | *** | 0.0000 |
| **Movement direction changes** | -0.13 | (0.87) |  | **1.15** | (1.13) | **** | 0.18 | (0.47) |  | 0.0000 |
| **Movement error** | -0.08 | (0.62) |  | 0.82 | (0.81) | **** | 0.50 | (0.45) | *** | 0.0000 |
| **Movement offset** | -0.04 | (0.42) |  | 0.30 | (0.55) | **** | 0.27 | (0.3) | ** | 0.0001 |
| **Movement variability** | -0.11 | (0.81) |  | **1.16** | (0.96) | **** | 0.66 | (0.58) | *** | 0.0000 |
| **Normalized jerk** | **1.46** | (1.39) | **** | **3.63** | (2.14) | **** | **1.66** | (1.18) | **** | 0.0000 |
| **Normalized jerk (w/o pauses)** | **1.38** | (1.21) | **** | **3.21** | (1.89) | **** | **1.55** | (1.17) | **** | 0.0000 |
| **Number of pauses** | 0.44 | (0.69) | **** | **1.22** | (0.72) | **** | 0.48 | (0.68) | * | 0.0000 |
| **Duration of the longest pause** | 0.42 | (0.66) | **** | **1.15** | (0.68) | **** | 0.46 | (0.66) | * | 0.0000 |
| **Orthogonal direction changes** | 0.05 | (1.06) |  | **1.57** | (1.07) | **** | 0.82 | (0.6) | **** | 0.0000 |
| **Peak speed** | -0.93 | (1.31) | **** | 0.52 | (1.72) | ** | 0.17 | (1.17) |  | 0.0000 |
| **Task axis crossings** | -0.25 | (0.77) | * | **1.13** | (1.06) | **** | 0.26 | (0.61) |  | 0.0000 |
| **Verification time** | 0.62 | (1.06) | *** | **1.15** | (1.22) | **** | 0.64 | (0.94) | * | 0.0095 |
| **Target reentries** | -0.14 | (0.7) |  | **1.14** | (1.1) | **** | 0.29 | (0.61) |  | 0.0000 |

Mean and standard deviation (SD) of z-scores for the Hevelius measures for participants with parkinsonism (n=46), ataxia (n=95), and the mild ataxia subset (n=16, BARS dominant arm score = 0). Asterisks next to a mean and standard deviation pair indicate that the z-score is significantly different from 0 (one-sample t-test, * p<.05, ** p<.01, *** p<.001, **** p<.0001). The last column indicates if the means are significantly different between ataxia and parkinsonism (independent samples t-test). Z-scores of 1.0 or greater in magnitude are **bolded**.

##

[**Table S3**](https://docs.google.com/document/d/1K8VOUHkE6cJGaQzppAadOowjay1SxcoIDG9k5K7Mukg/edit#table_classification). Results of classification analyses with age-specific vs generic z-scored features.

| **Comparison (N in parentheses next to each class)** | **Number of features used** | **Sensitivity** | **Specificity** | **Positive Predictive Value** | **Negative Predictive Value** |
| --- | --- | --- | --- | --- | --- |
| **Ataxia (68) Vs Parkinsonism (46)  (Age>=45)** | 11 | 0.897 | 0.891 | 0.924 | 0.854 |
| **Ataxia (68) Vs Parkinsonism (46)  (Age>=45, Generic z-scores)** | 24 | 0.794 | 0.783 | 0.844 | 0.720 |
| **Ataxia (21) vs Healthy (26) (Age <= 37)** | 2 | 0.857 | 0.923 | 0.900 | 0.889 |
| **Ataxia (21) vs Healthy (26)  (Age <=37, Generic z-scores)** | 3 | 0.905 | 0.846 | 0.826 | 0.917 |

The rows show the comparison of age-specific versus generic z-scores on classification performance for two different age groups. Age-matched cohorts were used for these analyses because generic z-scores capture a combination of age- and disease-related changes in motor performance.

[**Table**](https://docs.google.com/document/d/12WxREjGmj8B30XUAWRVU8a3Jm2pScAkSW-XXHSUHE1U/edit#table_combined_weights2) **S4.** The weights assigned to different features by the machine learning models (classification and regression models). Note that only a subset of the available features was used in any one model. Further, note that the magnitudes of the weights of the regression models are affected by the scoring scale and some weights were very small and therefore appear as “0.00” when rounded to two decimal places.

|  | **Ataxia-related analyses** | | | | **Parkinsonism-related analyses** | | | **Analyses related to both ataxia and parkinsonism** | | |
| --- | --- | --- | --- | --- | --- | --- | --- | --- | --- | --- |
| **Measure** | **Mild Ataxia (16) vs Healthy (29)** | **Ataxia (95) vs Healthy (29)** | **BARS dominant arm** | **BARS total** | **Parkinsonism (46) vs Healthy (29)** | **UPDRS dominant arm** | **UPDRS total** | **Ataxia (95) Vs Parkinsonism (46)** | **Common score dominant arm** | **Common score total** |
| **Peak acceleration** |  |  |  |  |  |  |  | -0.60 |  |  |
| **Peak speed** |  |  |  |  |  | -0.34 | -3.15 |  |  |  |
| **Noise to force ratio** | -1.30 |  |  |  | -1.70 | 0.35 | 0.75 |  |  |  |
| **Distance from target center at the end of the main submovement** |  |  |  |  |  |  | 1.04 |  |  |  |
| **Fraction of the distance to the target center covered in main submovement** |  |  | 0.06 | 0.48 |  |  | 1.63 |  | 0.01 | 0.02 |
| **Main submovement** | 1.30 | 1.80 |  |  | 0.00 |  |  | 1.30 |  |  |
| **Number of submovements** |  |  |  |  |  |  | 1.18 |  |  |  |
| **Max deviation from task axis** |  |  |  |  |  |  |  | 0.30 |  |  |
| **Movement error** |  |  |  |  |  |  | -0.26 |  |  |  |
| **Movement offset** |  |  |  |  | 1.00 |  |  |  |  |  |
| **Movement variability** |  |  |  |  |  | -0.42 |  | -0.50 |  |  |
| **Execution time** |  |  | 0.07 | 0.62 |  |  | 1.56 |  | 0.02 | 0.03 |
| **Execution time (w/o pauses) variability** | 0.80 |  |  |  |  |  |  |  |  |  |
| **Movement time** |  |  | 0.03 | 1.63 |  |  |  | -1.00 | 0.02 | 0.04 |
| **Normalized jerk** | -1.30 | -1.50 | 0.04 |  |  | 0.55 |  |  | 0.00 |  |
| **Normalized jerk (w/o pauses)** |  |  |  |  |  |  | -0.19 |  |  |  |
| **Execution time variability** | 0.60 | 1.10 |  |  |  |  |  |  |  |  |
| **Verification time variability** |  |  |  |  |  |  | 1.57 |  |  |  |
| **Movement direction changes** |  |  |  |  |  |  |  | -0.40 |  |  |
| **Target reentries** |  | -0.10 |  |  |  |  | 0.09 |  |  | 0.00 |
| **Task axis crossings** |  |  |  |  | 1.10 |  | -2.26 | -0.80 |  |  |
| **Click duration** |  |  | 0.10 |  |  | 0.19 | 0.59 |  | 0.02 |  |
| **Click duration variability** | 1.10 |  |  |  |  |  | 0.26 |  |  |  |
| **Click slip** |  |  |  | 0.19 | -1.50 | 0.34 | 2.76 | 0.90 |  | 0.01 |
| **Fraction of the main submovement spent accelerating** |  |  |  |  |  | 0.40 | 1.52 |  |  |  |
| **Peak acceleration variability** |  |  |  |  |  |  |  | 0.50 |  |  |
| **Peak speed variability** |  |  |  |  |  | 0.05 |  |  |  |  |
| **Verification time** |  |  |  | -0.26 |  | 0.37 |  | 0.30 |  | -0.00 |
